# Case of Cæsarean Section—Mother and Child Both Saved

**Published:** 1868-04

**Authors:** Wm. Warren Greene

**Affiliations:** Professor of Surgery in Berkshire Medical College, the Medical School of Maine, and in the University of Michigan; Pittsfield, Mass.


### Miscellaneous.

### Case of Cæsarean Section—Mother and Child both Saved.

By Wm. Warren Greene, M. D., Professor of Surgery in Berkshire Medical College, the Medical School of Maine, and in the University of Michigan.

On the 20th of August, 1867, I was called in consultation to see Mrs. B., aged 28, in her first labor, which began twenty-six hours previous to my arrival. Her physician, Dr. D. N. Emery, now of San Francisco, then spending the summer in Western Massachusetts, informed me that his patient had a pelvic deformity, which he feared would render delivery impossible, and he had for this reason called counsel. Upon examination I found the antero-posterior diameter of the superior strait less than two inches.*

Her pains were strong and frequent, and she began to exhibit marked symptoms of exhaustion, to which her consciousness of peril contributed not a little. The child was very active *in utero*. Upon explaining to herself and friends the probable impossibility of delivery *per vaginam*, and that even were there a bare chance of success by evisceration, she would, in her exhausted condition, incur greater risk from the operation under such circumstances than from abdominal section, the latter operation was assented to.

The case was so urgent as to admit of no delay, and we were therefore obliged to proceed with less assistance than I could have

* She had rachitis severely when a child.

desired. We were, however, fortunate in having the aid of Mr. Lewis Le Berne, of New York, a medical student, who happened to be in the neighborhood—a very intelligent and efficient man—and in addition we had the services of two of the most efficient ladies it has ever fallen to my lot to meet in the lying-in room—calm, self-possessed and intelligent.

The patient took a full dose of fluid extract of ergot with a little brandy, after which ether was administered. When under its influence, she was placed on a table, in the ordinary position for ovariotomy. I now, standing at her right, and while the abdomen was carefully supported on either side by assistants, with a common scalpel made an incision in the median line from a little above the umbilicus nearly to the pubes, which was soon carried through the abdominal walls and the uterus exposed. This organ was then incised from the fundus downward about six inches, the knife being used very cautiously until the cavity was opened and the *liquor amnii* evacuated. On carrying my right hand into the uterus, I readily seized the feet (which were on the left side, it being a vertex presentation,) and with little delay extracted the body, but some difficulty was experienced in delivering the head, occasioned by the powerful and unremitting uterine contractions, intensified, as I suppose, by the ergot. This, however, was soon accomplished, and the little fellow—a boy of eight pounds—cried lustily. Without waiting to sever the cord, an assistant supporting the child, I again introduced the hand in search of the placenta. This was attached on the left side about midway between the neck and fundus, and about one-third of it was detached. The remainder was readily separated, but its extraction, which was soon accomplished, with the membranes, was by no means an easy task. I had not anticipated so powerful muscular action in an organ thus mutilated.

There was considerable hæmorrhage during the delivery, but not sufficient to cause any serious apprehension, and it ceased at once upon the removal of the placenta, the edges of the uterine wound being nicely approximated by the contractions of that organ. Unquestionably the ergot had fulfilled the indication for which it was given, namely, to control hæmorrhage and secure apposition of the cut edges by its action upon the uterine muscular fibres.

After carefully cleansing the parts with sponges dipped in water at blood heat, and then thoroughly moistening them with artificial serum at the same temperature, the external wound was closed by interrupted sutures placed half an inch apart, and including the entire thickness of the parietes except the peritoneum. These were of silk soaked in boiling wax, as we had no silver wire at hand, a fact that caused me not a little anxiety at the time, although I may say, not only from its use in this but in many other instances, that smooth, well-twisted silk sutures thus prepared approximate very closely in value to those of silver.

The abdomen, which had been unremittingly supported by the hands, was now enveloped in a firm bandage, and the woman put in bed well covered, and dry heat applied to the extremities, which were rather cool. They soon became warm, however, and as soon as she could swallow she got twenty-five drops of fluid extract of etgot and half a grain of morphia. After the effect of the ether had passed away, the pulse was over 100 and rather feeble. Countenance pale, with that peculiar expression which indicates a marked shock. She was rather restless and *wakeful.* She now got morphia and brandy, with beef-juice, and from 6 P. M. till 3 A. M., she took one grain of morphia and *one quart* of brandy. (This amount of morphia in addition to the half grain which she took at 5 o'clock, just after the operation.) Just after 3 A. M., she fell into a quiet sleep, which lasted five hours, from which she awoke in excellent condition.

The treatment now instituted was perfect quiet; anodynes *pro re nata*, ten drops of fluid extract of ergot and twenty-five drops of tincture of muriate of iron every four hours, the two alternating—the former to be omitted in forty-eight hours and the latter to be continued, if borne by the stomach, until the external wound was healed.

The farther history of the case contains nothing of special interest. The external wound healed throughout by first intention. A moderate peritonitis followed, but not sufficient at any time to require *heroic* doses of opium. The iron was well borne throughout, and the lochial discharge occurred and continued as after an ordinary case of labor.

In a letter dated August 30th, (tenth day after the operation,) Dr. Emery says:—"Have just returned from Oak Hill, and am happy to report Mrs. B. in fine condition. I have removed the last stitch. There is very little fulness or tenderness of the bowels." The mother and child are now in excellent health.

Too much praise cannot be bestowed upon physician and nurses for the skillful and careful after-treatment of this case, and especially to Mr. Berne, who hardly left the bed-side for a week after the operation.

Before closing this paper, I cannot forbear saying a word about that old-fashioned and somewhat homely remedy, the muriated tincture of iron. While all members of the profession admit its virtue as a restorative hæmatic in some degree, yet I believe very few are aware with what *rapidity* and *certainty* it increases the plasticity of the blood. Why the difference I do not know, but I feel very sure that no other chalybeate preparation is to be compared with it for this purpose. Given for a time previous to, or immediately after operations, as a prophylactic against erysipelas, phlebitis, ulceration, secondary hæmorrhage, etc., it is invaluable. But it must be borne in mind that for a decided and rapid impression, *large* doses are requisite, or smaller ones given very frequently; and these doses are usually well borne if care is taken to *dilute* and *sweeten* it thoroughly. It can thus be made for a patient a little thirsty a comparatively pleasant drink. My friend, Prof. E. Andrews, of Chicago, some years ago called the attention of the profession to these facts, and it gives me pleasure to corroborate his views and statements from my own experience.

Pittsfield, Mass., Nov. 5, 1867.

